# Development of a Rapid UHPLC-PDA Method for the Simultaneous Quantification of Flavonol Contents in Onions (*Allium cepa* L.)

**DOI:** 10.3390/ph14040310

**Published:** 2021-04-01

**Authors:** Ana V. González-de-Peredo, Mercedes Vázquez-Espinosa, Ceferino Carrera, Estrella Espada-Bellido, Marta Ferreiro-González, Gerardo F. Barbero, Miguel Palma

**Affiliations:** Department of Analytical Chemistry, Faculty of Sciences, Agrifood Campus of International Excellence (ceiA3), University of Cadiz, IVAGRO, Puerto Real, 11510 Cadiz, Spain; ana.velascogope@uca.es (A.V.G.-d.-P.); mercedes.vazquez@uca.es (M.V.-E.); ceferino.carrera@uca.es (C.C.); estrella.espada@uca.es (E.E.-B.); marta.ferreiro@uca.es (M.F.-G.); miguel.palma@uca.es (M.P.)

**Keywords:** *Allium cepa* L., Box–Behnken, flavonoids, quercetin glycosides, liquid chromatography, multiresponse optimization, onion, phenolic compounds, UHPLC

## Abstract

Onion, one of the most consumed vegetables in the world, is also known to contain high levels of antioxidant compounds, with protective effects against different degenerative pathologies. Specifically, onion is rich in flavonols, mainly quercetin derivatives, which are compounds with high antioxidant and free radical scavenging power. For this reason, it is of the utmost importance to count on optimal analytical methods that allow for the determination and quantification of these compounds of interest. A rapid ultra-high performance liquid chromatography (UHPLC)-photo-diode array (PDA) method for the separation of the major flavonols in onions was developed using a Box–Behnken design in conjunction with multiresponse optimization on the basis of the desirability function. The conditions that provided a successful separation were 9.9% and 53.2% of phase B at the beginning and at the end of the gradient, respectively; 55 °C column working temperature; and 0.6 mL min^−1^ flow rate. The complete separation was achieved in less than 2.7 min with excellent chromatographic characteristics. The method was validated, and its high precision, low detection and quantification limits, good linearity, and robustness were confirmed. The correct applicability of the method improves the analysis of the raw material, increasing the quality of onions and its subproducts in terms of bioactive compounds and functional characteristics for consumers.

## 1. Introduction

Onion (*Allium cepa* L.) is one of the most widely grown and consumed vegetables in the world [1]. This vegetable has shown a constant increasing production trend by more than 25% in the past few years [2]. Onion’s pungent and unique taste makes it an ideal food condiment that is highly appreciated by consumers, favoring its trend increase. However, this increasing production tendency is also supported by the current awareness by consumers of its high content in non-nutrient bioactive compounds with preventive properties against some degenerative pathologies [3,4]. Specifically, onion presents antimicrobial, antioxidant, anticarcinogenic, antimutagenic, antiasthmatic, immunomodulatory, and cardiovascular properties [5,6]. The flavonols present in onions are some of the main contributors to these health-promoting properties. Every single type of onion (white, yellow, or red) is rich in flavonols, mainly quercetin derivatives, being recognized as one of the major dietary sources for quercetin [7]. Quercetin is a very interesting flavonol because of its antioxidant and free radical scavenging power, which gives onions their ability to protect against multiple diseases [8]. It should be highlighted that a recent research study has identified the natural compound quercetin as an inhibitor of severe acute respiratory syndrome coronavirus (SARS-CoV-2 3CLpro), one of the essential proteases for the replication of the coronavirus known as COVID-19 [9]. Specifically, quercetin has shown plenty of desirable characteristics and potential (inhibition constant Ki ~ 7 μM), allowing it to be considered an attractive candidate for further optimization and development in this field. With regards to onions, quercetin 3,4′-*O*-diglucoside and quercetin 4′-*O*-glucoside are the primary glucosides found in onion bulbs [10] since they account for about 95–99% of their total flavonol content [11]. The structures of these two particular flavonols and the rest of the flavonols present in onions can be referred to in Figure S1. A detailed analysis of these compounds is of great interest since it would allow for the evaluation of this vegetable’s metabolomic profile. For this reason, it is of the utmost importance to count on optimal analytical methods that allow for the determination and quantification of these compounds of interest. Different techniques have been primarily employed for the determination of flavonols in onions. The analytical platform of choice for this study was ultra-high performance liquid chromatography (UHPLC) coupled to a photo-diode array (PDA). UHPLC represents a further advance in chromatographic techniques with additional advantages such as high-resolution separations using under 2 µm diameter solid-phase particles [12]. This implies high resolution and sensitivity in extremely short run times and, therefore, a lower cost on solvents [13]. Thus, UHPLC stands at a leading position for the analysis of phenolic compounds, and it has been widely used in numerous research studies on the phenolic contents that can be found in numerous natural matrices [14]. Regarding the detectors, as aforementioned, in this study, UHPLC system was coupled to a photo-diode array (PDA). PDA is one of the most commonly used detectors because of the wide range of molecules that can be analyzed, its relatively low price, and its availability in most food analysis laboratories. Specially this work wants to highlight the importance of using the PDA as an affordable detector for laboratories and food and quality industries. Most articles found in the literature use UHPLC coupled to mass spectrometry [15,16]. Mass spectrometry presents some substantial advantages, such as very high sensitivity and data collection on molecular mass and structural characteristics, but it is not always available to research groups or company laboratories in general because of its high costs. Furthermore, chromatographic methods that can be found in the literature require over 30 min to complete the successful separation of all the peaks [10,17,18]. In this work, the aim was to achieve the analysis of the main compounds of interest in minimum times, less than 5 min, separations that are difficult to achieve with mass spectrometry. This time reduction is an essential factor for any analysis procedure, and it is particularly interesting when intended to be used at an industrial scale, where time is critical.

According to some of the previous works published by our own research group [19,20], the use of UHPLC, without mass spectrometry and combined with a correct optimization of the chromatographic characteristics (solvent flow, gradient, etc.), allows for a successful analysis in a considerably shorter time. Analysis time and peak resolution are the two most important aspects to be taken into consideration for the optimization of a chromatographic method [14]. Both parameters depend mainly on gradient time and flow rate, and thus their influence on a successful separation is generally evaluated. In this work, it is intended to make use of the experiment designs combined with UHPLC for the study of flavonols in onion. Response surface methodology (RSM) is an excellent tool for studying and optimizing these variables. The use of RSM represents further advantages compared to conventional optimization methods in which only one variable is studied at the same time. Specifically, RSM allows for studying several variables and knowing both their individual and combined effect on the response variable, without the need to carry out a large number of experiments. This makes it a very useful tool without the need to involve high manufacturing costs [21]. Among the different RSM options, the Box–Behnken design (BBD) is often used for the statistics study, since a shorter number of runs is required when compared to other methodologies. This design saves time and avoids running experiments under extreme conditions, which might pose undesirable risks with regards to the reliability of the results. On the other hand, when the optimization procedure involves more than one response, each one of them cannot be optimized separately, since a number of solutions equal to the number of variables under study would be generated [20]. In such cases, a multiresponse optimization (MRO) with desirability functions is usually employed, which has been proven to be an effective statistical tool to solve multi-variable problems and to optimize either single or multiple responses [22]. Specifically, it has been proved to be useful for developing, improving, and optimizing processes, which was the objective of this study.

Therefore, the present work intended to develop and validate a rapid and reliable UHPLC-PDA methodology for the simultaneous separation of the flavonols present in onions by means of a BBD in conjunction with MRO and desirability functions. This method would allow laboratories, researchers, and companies to analyze the main flavonols found in onions without having to use mass spectrometry, doing so in a short time. A successful applicability of this methodology would improve analysis procedures and, therefore, would also favor the final quality of onions and their subproducts.

## 2. Results and Discussion

### 2.1. UHPLC Acquisition of the Responses

A BBD design was employed to determine the effect of three factors (the three in-dependent variables) on the UHPLC-PDA separation. The factors studied were flow rate (X_1_), solvent composition (% phase B) at the beginning of the gradient program (X_2_), and solvent composition (% phase B) at the end of the gradient program (X_3_). Specifically, the conditions studied for each variable were flow rate (mL min^−1^): 0.4, 0.5, 0.6; %B at the beginning (%): 0, 5, 10; and %B at the end of the gradient program (%): 50, 75, 100. The onion extract was injected into the UHPLC system according to the BBD experiments (Table 1). The 15 experiments were carried out independent and randomly, while the values of the independent variables were the only variations between each other.

With respect to the response variables (dependent variables), these were optimized according to the peak resolutions (Y_Rs_), and the analyses run times (Y_RT_). Resolution (R_S_) is a numeric value that indicates how much a peak overlaps the adjacent peak from a perpendicular line crossing the trough. Most studies set a minimum resolution target of around 1.5 for a complete peak separation [23]. Resolution values of 1 imply a 4% overlap between the two adjacent peaks, while values of 1.5 result in an overlap of only 0.3%. Any value lower than 1 indicates a poor separation. In addition to the R_S_, analysis run time (RT) was also considered as a variable response for the method optimization. This variable is related to the retention time of the final peak in the UHPLC chromatogram. Therefore, the final objective of the experimental design was to achieve maximum resolutions that did not imply exceedingly long analysis times. Among the seven major flavonols identified in the onion, quercetin 3,7,4′-*O*-triglucoside (peak 1) and quercetin 7,4′-*O*-diglucoside (peak 2) were discarded for the experimental design, since their peaks presented short retention times and therefore did not influence the analysis run time while achieving excellent separation in the 15 experiments that were carried out. Consequently, the resolutions of each one of the five major peaks identified (peak 3, peak 4, peak 5, peak 6, and peak 7) were considered for response variable optimization purposes. In particular, the four R_S_ values in this category were R_S3–4_, R_S4–5_, R_S5–6_, and R_S6–7_. The number corresponds to the elution order of the compounds in the column. The resolutions and the analysis run time resulting from each BBD experiment are shown in Table 1.

### 2.2. Optimization of the UHPLC Method

The MRO was used to determine the optimum chromatographic conditions for all the responses at the same time. Specifically, the effect of the three chromatographic working variables on the five responses were evaluated. Previous to this MRO, a RSM was employed to generate a separate model for each response. Then, the desirability function was constructed on the basis of the values obtained for each optimized response.

A total of five second-order mathematical models that represent the correlation between each independent variable and each response were generated. The resulting equations (Equations (1)–(5)) for the fitted models are as follows:

***Y*_Rs3−4_** = 1.56667 + 0.075***X*_1_**+ 0.2125***X*_2_** − 0.4625***X*_3_** − 0.120833***X*_1_^2^** + 0.05***X*_1_*X*_2_** + 0.05***X*_1_*X*_3_** − 0.045833***X*_2_^2^** − 0.125***X*_2_*X*_3_** + 0.104167***X*_3_^2^** (1)

***Y*_Rs4−5_** = 3.9 + 0.25***X*_1_**+ 0.45***X*_2_** − 0.625***X*_3_** − 0.1375***X*_1_^2^** + 0.075***X*_1_*X*_2_** + 0.125***X*_1_*X*_3_** + 0.0125***X*_2_^2^** − 0.125***X*_2_*X*_3_** − 0.0875***X*_3_^2^** (2)

***Y*_Rs5−6_** = 3.43333 + 0.275***X*_1_**+ 0.2375***X*_2_** − 0.8375***X*_3_** − 0.0666667***X*_1_^2^** + 0.025***X*_1_*X*_2_** − 0.325***X*_1_*X*_3_** − 0.0916667***X*_2_^2^** + 0.05***X*_2_*X*_3_** + 0.158333 ***X*_3_^2^** (3)

***Y*_Rs6−7_** = 1.7 + 0.15***X*_1_**+ 0.1125***X*_2_** − 0.6125***X*_3_** + 0.0***X*_1_^2^** + 0.025***X*_1_*X*_2_** − 0.175***X*_1_*X*_3_** + 0.025***X*_2_^2^** − 0.05***X*_2_*X*_3_** + 0.225***X*_3_^2^** (4)

***Y*_RT_** = 2.765 − 0.183125***X*_1_** − 0.29425***X*_2_** − 0.442875***X*_3_** + 0.043***X*_1_^2^** − 0.01425***X*_1_*X*_2_** + 0.0105***X*_1_*X*_3_** − 0.02375***X*_2_^2^** + 0.08175***X*_2_*X*_3_** + 0.1315***X*_3_^2^** (5)

The different polynomial equations can be used to know what the value of the response variable will be if the value of the dependent variables is known. The greater the efficiency of these equations, the higher the correlation coefficient (*R*^2^). The coefficients obtained ranged from 82.68% for R_S4–5_ to 99.94% for run time, which indicate a statistically significant agreement between the measured and the estimated responses.

**Figure 1:**
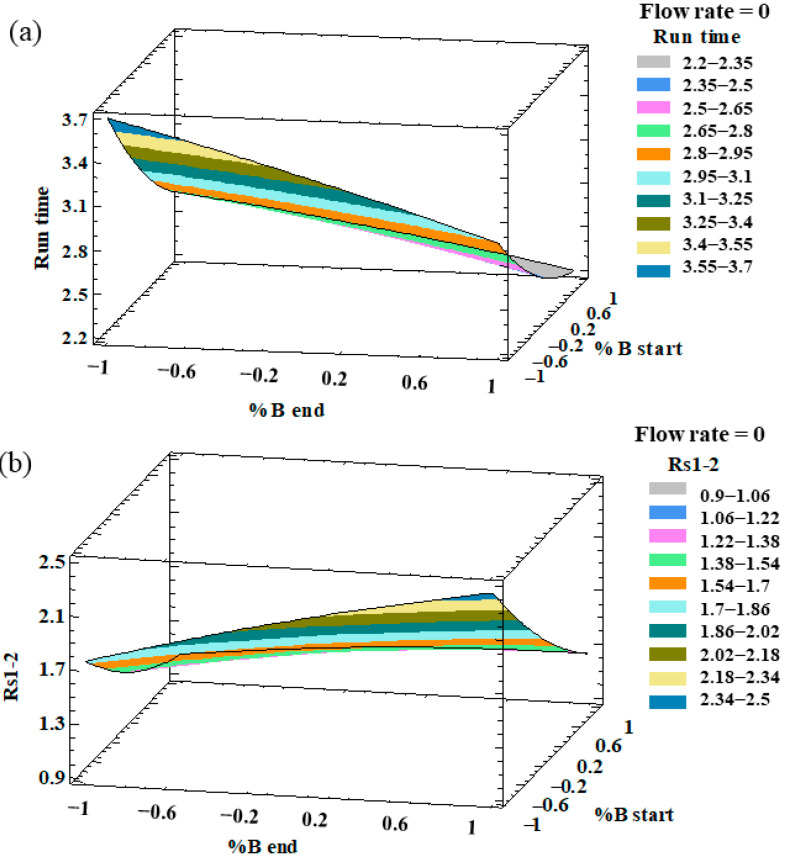
3D surface plots of the Box–Behnken design to represent the influence of (**a**) %B at the end of the gradient–%B at the beginning of the gradient on the run time; (**b**) %B at the end of the gradient–%B at the beginning of the gradient on the resolution of peaks 1 and 2 (R_S1–2_).

An analysis of variance (ANOVA) was individually applied to evaluate the effect of the different factors on each response and the possible interactions between them. The factors and/or interactions that showed a *p*-value lower than 0.05 were considered to be significant factors with an influence on the response at the established level of significance (95%). With regards to the resolutions, all the responses showed *p*-values lower than 0.05 for percentage of phase B at the beginning of the gradient, which indicates that this factor had a significant effect on all the resolution responses. In particular, this factor had a negative effect, which means that the peak resolutions increased with a low %B at the beginning of the gradient. On the other hand, the percentage of phase B at the end of the gradient presented a positive relevant effect on both R_S3–4_ and R_S4–5_, with a *p*-value < 0.05. With regard to the analysis run time, the three factors (flow rate, %B at the beginning, and %B at the end) had *p*-values lower than 0.05. The optimization target for this response variable was to minimize the analysis run time. In this sense, and on the basis of the ANOVA results, we concluded that in order to minimize the analysis time, a greater flow rate, a greater %B at the beginning of the gradient, and a greater %B at the end of the gradient, were necessary. Therefore, a high %B at the end of the gradient and a high flow rate favored the separation of the peaks and decreased the analysis time. On the other hand, the %B at the beginning had the opposite effect on the resolutions and run times. Such an opposite trend can be graphically observed in the three-dimensional (3D) surface plots that have been generated from the fitted model. The combined effects of %B at the end-%B at the beginning on the run time and R_S1–2_ are represented in Figure 1.

Subsequently, the MRO was used for the simultaneous optimization of the six responses. All the responses were considered to be equally important chromatographic characteristics for the separation and determination of the flavonols. The weight of each response in the computational analysis was determined by the impact coefficient that had been assigned to each response by the MRO. The run times obtained in the 15 BBD experiments varied between 2.210 and 3.699 min. This run time range was considered as acceptable for rapid separations, especially when compared to the analysis run times of the UHPLC methods for onion flavonols published in the literature. Although some methods require a time as relatively short as 15 min [24,25], most of them require at least 30 min to complete the correct separation of all the peaks [6,11], and some of them may even need as much as 60 min [17,18]. However, the impact for this variable was set to the highest relevance (5 impact), since one of the main objectives of this work was to develop a time-and-cost-saving multiple analysis UHPLC methodology. With regards to the resolutions, all the R_S4–5_ (ranging from 2.46 to 5.40) and R_S5–6_ (ranging from 2.12 to 4.91) obtained from the 15 BBD experiments were higher than 1.5, which is the minimum resolution required for a successful and complete separation. Therefore, the impact of these variables was set as lower importance (3 impact). Finally, R_S3–4_ (ranging from 1.00 to 2.35) and R_S6–7_ (ranging from 1.22 to 2.92) with resolutions under 1.5 in some of the BBD experiments were set at an intermediate relevance level (4 impact). Thus, the optimization defined by MRO was to maximize the resolution and minimize the run time.

**Figure 2:**
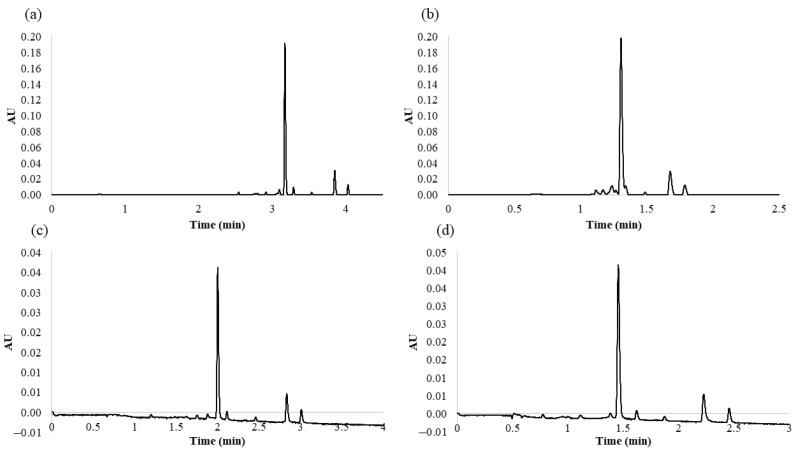
Different chromatograms obtained under different conditions: (**a**) chromatogram obtained from the 2° Box–Behnken design (BBD) experiment (analysis time longer than the established optimal conditions); (**b**) chromatogram obtained from the 11° BBD experiment (overlapping peaks); (**c**) chromatogram obtained at the lowest temperature, 35 °C (analysis times longer than the established optimal conditions); (**d**) chromatogram obtained at the highest temperature, 65 °C (poorer resolution than under the established optimal conditions).

From the MRO design, it was possible to extract information about the optimum values which show the maximum response for all the variables, that is, optimize the desirability function. Therefore, according to the MRO, the optimal values for the studied variables were as follows: 9.9% for %B at the beginning of the gradient, 53.2% for %B at the end of the gradient, and 0.6 mL min^−1^ for the flow rate. With regards to the flow rate, no higher flows were tested since they would imply really high pressure within the system. Thus, the maximum flow rate was restricted by the system pressure limit at 15,000 psi. The analysis time between the beginning of the gradient and the end of the gradient was 5 min. With these settings, the response variables generated a desirability index of 86.48%. For comparative purposes, Figure 2a,b shows the chromatograms obtained under a series of other conditions according to the BBD experiments.

**Figure 3:**
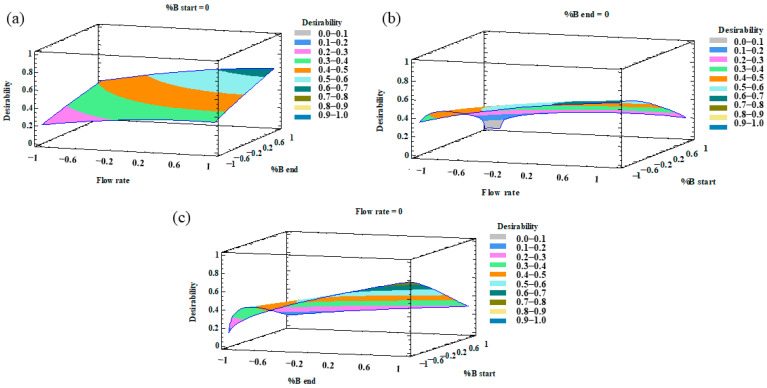
3D surface plots of the multiresponse optimization (MRO) design representing the influence on the desirability function by (**a**) flow rate–%B at the end; (**b**) flow rate–%B at the beginning; (**c**) %B at the end–%B at the beginning.

Once the MRO had been performed, the desirability was plotted as a 3D contour plot that illustrates the result of the optimization of all the variables. The combined effects of %B at the beginning–%B at the end, %B at the start-flow rate, and %B at the end-flow rate are represented in Figure 3.

Once MRO had been applied, the effect that the column temperature had on the separation of the flavonols was also evaluated. For this purpose, and on the basis of the principles of column temperature changes, we gradually increased the temperature from 35 to 65 °C in 5 °C intervals in order to evaluate the effect of different column temperatures. Higher temperatures beyond the above-mentioned range were not tested, since according to Waters Corporation’s recommendation, it may result in a shorter column lifetime. To compare the effect of the temperature on the separation of the five major compounds identified in the onion matrices, we evaluated peak resolution and analysis time. As the temperature was increased, the analysis time decreased. Higher temperatures had a positive effect, since they reduced the viscosity of the mobile phase, which in turn decreased column pressure and the compounds eluted at a greater speed (temperature (°C), t_R_ (min): 35, 3.01; 40, 2.92; 45, 2.83; 50, 2.71; 55, 2.65; 60, 2.54; 65, 2.46). These results confirmed that the method was more efficient at the highest temperatures within the studied range (55, 60, and 65 °C). Among these temperatures, 55 °C seemed to provide the best results in the separation of the seven major compounds identified in the red onion samples, since it resulted in narrow and very well resolved peaks in a very short run analysis time. At 60 and 65 °C, most of the peak chromatogram resolutions decreased (temperature (°C), resolution (R_S4–5_): 55, 5.29; 60, 4; 65, 4.19); (temperature (°C), resolution (R_S5–6_): 55, 5.36; 60, 4.28; 65, 4.83) due to the shortening of the total run analysis time. The chromatograms obtained at the lowest (35 °C) and highest (65 °C) temperatures within the range are shown in Figure 2c,d, respectively, to clearly observe the results previously described.

### 2.3. Characteristics of the Developed Method

**Figure 4:**
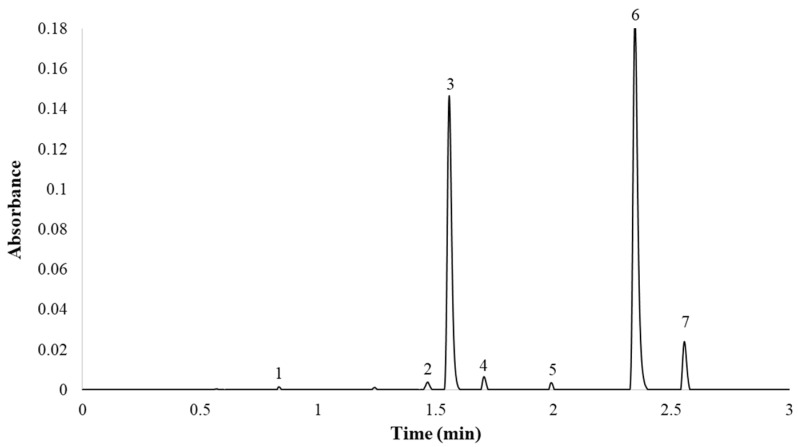
Red onion chromatogram at 360 nm. (1) Quercetin 3,7,4′-*O*-triglucoside; (2) quercetin 7,4′-*O*-diglucoside; (3) quercetin 3,4′-*O*-diglucoside; (4) isorhamnetin 3,4′-*O*-diglucoside; (5) quercetin 3-*O*-glucoside; (6) quercetin 4′-*O*-glucoside; (7) isorhamnetin 4′-*O*-glucoside.

Finally, the optimum gradient of the UHPLC-PDA method developed in this study was as follows: 0.0 min, 9.9% B; 5.0 min, 53.2% B; 5.10 min, 100% B; 7.0 min, 100% B; 7.5 min, 9.9% B; 10 min, 9.9% B. The column temperature was maintained at 55 °C and the flow rate was 0.6 mL min^−1^. The total analysis time (sample-to-sample) was 10.0 min, including the return to the initial conditions and the re-equilibration of the column, while the separation of the seven major compounds was completed in less than 2.7 min. A representative chromatogram employing PDA (λ = 360 nm) is presented in Figure 4.

It can be seen from the results that the compounds were correctly separated, with narrow peaks in a very short time. Thus, the selected conditions provided the best balance between reduced analysis time and successful separation of the seven peaks. Although there were articles in the bibliography that identified a greater number of compounds in onion, this work focused on the analysis of the seven onion flavonol majority. Only quercetin 3,4′-*O*-diglucoside and quercetin 4′-*O*-glucoside represented around 95–99% of the total flavonol content. This work aimed to provide an analytical utility for industries, wherein analyzing in less than 3 min the almost total percentage of flavonols is of greater interest than the analysis of minorities (<10%).

The chromatographic properties evaluated were retention time (t_R_), selectivity (α), retention factor (*k*), and resolution (Rs). The chromatographic properties obtained with the developed method are shown in Table 2.

On the basis of the results obtained, we were able to conclude that the developed UHPLC method provides a successful separation of the chromatographic peaks with excellent resolutions, retention factors, and selectivities. Regarding the resolutions, all the values obtained were higher than 1.5, which implies an overlap of the peaks lower than 0.3%. With regards to the retention factor (*k*), 1 < *k* < 10 is usually the target range [26]. The retention factor is a measure of the time that a compound remains in the stationary phase relative to the time that it remains in the mobile phase. Therefore, values greater than 10 do not imply a significant resolution increment but lead to excessively long retention times. Values significantly lower than 1 indicate that the analyte leaves the column when close to the dead time. In the case of this work, all the retention factor values were within the target range. Finally, in relation to selectivity, α > 1 is usually the target range. A high α value indicates a clear separation between the peaks. In the case of this work, all the selectivity values were greater than 1.

### 2.4. Validation of the Developed Method

The developed method was validated in accordance with ICH Guideline Q2 (R1) [27]. Linearity, precision, limit of detection, and quantification as well as robustness were evaluated. The validation results are reported in Table 3 and Table 4.

The linearity of the method was confirmed by the regression coefficient for quercetin 3-*O*-glucoside (r = 0.9997), which was obtained from the calibration curve of the commercially available standard constructed using six points (0.1–200 mg L^−1^) in triplicate. The calibration curves for the rest of the compounds were estimated on the basis of the curve for quercetin 3-*O*-glucoside and each compound’s molecular mass ratio and assuming that the seven flavonols have similar absorbance because of their similar chemical structures. This is a usual quantification procedure when the standard for any of the compounds of interest is not commercially available [14]. The *R*^2^ showed values close to 1, which indicates that all the compounds identified in the onion matrices presented a good linearity within the target range (Table 3).

The repeatability and intermediate precision of the developed methodology were determined according to both retention time and chromatographic resolution. With respect to retention time, its repeatability and intermediate precision showed coefficients of variance (CV) lower than 3% for all the peaks. With regards to resolution, its repeatability and intermediate precision resulted in coefficients of variance (CV) lower than 5% for all the peaks (Table 3). It can be confirmed that in all the cases the CVs were within the acceptable limits (±10%) according to Association of Official Agricultural Chemists (AOAC) [28], and therefore support the accuracy of the developed UHPLC methodology.

The limit of detection (LOD) and limit of quantification (LOQ) for quercetin 3-*O*-glucoside were estimated as 3 and 10 times the signal-to-noise ratio, respectively. The LODs and LOQs of the rest of the compounds were calculated according to the corresponding values for quercetin 3-*O*-glucoside as well as each compound’s molecular mass ratio. The LODs ranged from 0.0257 to 0.0437 mg L^−1^ and the LOQs ranged from 0.0857 to 0.1454 mg L^−1^ (Table 3).

Finally, the robustness of the method (Table 4) was evaluated by testing specific variations in flow rates, injection volumes, and column temperatures. Each parameter was tested at three different levels, and for each level, four repetitions were carried out. The effect of these variations on the retention times, the chromatographic resolution of the peaks, and the area of the chromatographic peaks were verified. The robustness results are reported in Table 4, where different letters (a, b, and c) in the same row indicate that significant differences were detected according to the *t*-test assuming equivalent variances (*p*-value < 0.05). No significant differences (*p*-values > 0.05) were found between the peak resolutions and areas resulting from the controlled variations, which allowed us to conclude that the controlled variations in temperature, flow rate, or injection volume did not give place to any relevant influences on peak resolution or area. The methodology was also proven to be robust (*p* > 0.05) with regards to retention time when the injection volume was varied. However, some of the compounds’ retention times presented statistically relevant differences when the flow rate or the temperature were modified. This was to be expected, since retention time depends not only on the polarity of the specific molecule, but also on factors such as flow rate and temperature. For example, at a higher flow rate, the component molecules had less time to interact with the stationary phase as they were quickly pushed through the column. It is, therefore, necessary to keep an adequate control of the flow rate and the temperature by a proper balancing and conditioning of the equipment to provide an accurate resolution and a clear separation of the flavonols’ peaks.

### 2.5. Application of the Developed Method to Different Onion Varieties

**Figure 5:**
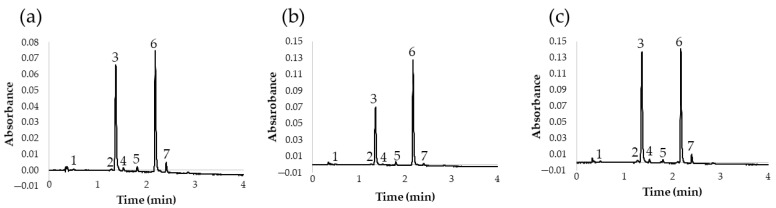
Chromatogram at 360 nm of different onion varieties: (**a**) white onion; (**b**) yellow onion; (**c**) red onion. (1) Quercetin 3,7,4′-*O*-triglucoside; (2) quercetin 7,4′-*O*-diglucoside; (3) quercetin 3,4′-*O*-diglucoside; (4) isorhamnetin 3,4′-*O*-diglucoside; (5) quercetin 3-*O*-glucoside; (6) quercetin 4′-*O*-glucoside; (7) isorhamnetin 4′-*O*-glucoside.

Once the UHPLC-PDA method for the analysis of flavonols in red onion had been optimized, an additional study was carried out to determine the flavonol content in different types of onions. This should demonstrate the applicability of the method to the analysis of different onions with different chemical compositions. Specifically, 13 onion varieties were studied: 6 white onions, 3 yellow onions, and 4 red onions. The results of the analyses are shown in Table 5 and the chromatograms obtained for a yellow, a red, and a white variety are also shown in Figure 5.

According to these results, we can conclude that the proposed UHPLC method was adequate for the analysis of different onion varieties, since it presented good chromatographic characteristics and short run times. This is of great interest, among other things, because it allows for the characterization of onion varieties according to their flavonol profile in a short time and with a high precision level. In fact, according to the resulting quantifications of flavonoids, it could be concluded that the major flavonols in red, yellow, and white varieties were quercetin 3,4′-*O*-diglucoside and quercetin 4′-*O*-glucoside, representing about 90% of their overall flavonol content. Furthermore, according to these results, the content of total flavonoids is considerably higher in red onions. Specifically, the red varieties presented nearly double flavonoid contents when compared to yellow or white onions. These results are in agreement with those already reported by other authors, who recommend the consumption of red varieties because of their greater flavonoid contents and health benefits [29]. On the other hand, flavonoid contents in white and yellow varieties were found to be very similar, and minor differences were mostly due to either specific variety (sweet, spring, etc.) or geographical origin.

## 3. Materials and Methods

### 3.1. Chemicals and Reagents

The solvent used for onion extraction was a mix of methanol and water. The methanol (Fischer Scientifics, Loughborough, United Kingdom) was HPLC-grade. The ultra-pure water was obtained from a Milli-Q water purification system (EMD Millipore Corporation, Bedford, MA, USA). For UHPLC analyses, acetonitrile (Panreac, Barcelona, Spain), water, and acetic and formic acid (Merck KGaA, Darmstadt, Germany) were used. The solvents were degassed and filtered through a 0.22 µm membrane (Nylon Membrane Filter, FILTER-LAB, Barcelona, Spain) before being used. The standard for the quantification of flavonols was quercetin 3-*O*-glucoside supplied by Sigma-Aldrich (Steinheim, Germany).

### 3.2. Plant Material

The plant material employed for the development of the method consisted of samples from red onion purchased from a local market in the province of Cadiz (Spain). The onions were subjected to a pretreatment in order to improve sample-solvent contact surface [30]. First, the onion bulbs were washed, cleaned, peeled, and cut using a knife. Then, the chopped bulbs were lyophilized in a freeze dryer LYOALFA (Azbil Telstar Technologies, Terrassa, Barcelona, Spain) and crushed in a knife mill GRINDOMIX (Retsch GM200, Haan, Germany). Furthermore, other different onion varieties, purchased from local markets in the province of Cadiz (Spain) and subjected to the same pretreatment, were also analyzed. Specifically, 6 white onion (spring and sweet), 3 yellow onion, and 4 red onion varieties were studied. Information about the characteristics of the different onions (origin, producer, caliber, etc.) are included in the Supplementary Materials (Table S1). Finally, all the samples were stored in a freezer at −20 °C.

### 3.3. Extraction Procedure

To extract the flavonols from the onions, we used ultrasound-assisted extraction. Specifically, a Sonopuls HD 2070.2 probe (BANDELIN electronic GmbH and Co KG, Heinrichstrabe, Berlin, Germany) was employed, coupled to a processor for amplitude (80% of the maximum amplitude) and cycle (0.5 s) adjustments. For temperature control (20 °C), a thermostatic bath (Frigiterm-10, Selecta, Barcelona, Spain) was employed. About 0.2 g of the lyophilized and homogenized onion was weighed into a Falcon tube, and 20 mL of 50:50 MeOH/H_2_O mixture was added. The Falcon tube was placed in a double vessel through which the water from the thermostatic bath circulated. The extraction time was set at 10 min. After this time, the extracts were centrifuged at 7500 rpm (9.5 cm orbital radius, 5 min) and the supernatants were placed into a volumetric flask of 25 mL. The precipitates from the extraction were redissolved in 5 mL of the same extraction solvent and centrifuged again under the same conditions. The new supernatants were placed into volumetric flasks and topped up with the same solvent. The final extracts were stored at −20 °C for their correct conservation until further analysis. The ultrasound-assisted extraction (UAE) conditions that were set for the extractions were based on previous results obtained by our research group for phenolic compounds [31,32].

### 3.4. Identification of Flavonols by Liquid Chromatography Coupled to Mass Spectrometry

Because not all the standards were available for the group, we carried out a previous identification using liquid chromatography coupled to mass spectrometry. Specifically, the major flavonols present in *Allium cepa* L. were identified by ultra-high performance liquid chromatography (UHPLC) coupled to a photodiode array (PDA) detector (Waters Corporation, Milford, MA, USA) and a quadrupole-time-of-flight mass spectrometer (Q-ToF-MS) (Xevo G2 QToF, Waters Corp., Milford, MA, USA).

The column was a reverse-phase C18 analytical column with 1.7 μm particle size, 2.1 × 100 mm (ACQUITY UPLC CSH C18, Waters). The mobile phase A, 2% formic acid–water solution, and the mobile phase B, 2% formic acid–methanol solution, were used at a flow rate of 0.4 mL min^−1^. The gradient employed was the following (time, % solvent B): 0.00 min, 15%; 3.30 min, 20%; 3.86 min, 30%; 5.05 min, 40%; 5.35 min, 55%; 5.64 min, 60%; 5.95 min, 95%; and 7.50 min, 95%. The total run time was 12 min: 8 min for the analysis and 4 additional minutes for re-equilibration. The mass spectra were acquired in negative ion mode under the following conditions: desolvation gas flow = 700 L h^−1^, desolvation temperature = 500 °C, cone gas flow = 10 L h^−1^, source temperature = 150 °C, capillary voltage = 700 V, cone voltage = 30 V, and collision energy = 20 eV. The ions were scanned from *m/z* 100 to *m/z* 1200.

Prior to their identification, all the UAE extracts were filtered through a 0.20 µm nylon syringe filter (Membrane Solutions, Dallas, TX, USA), and 3 µL was the volume injected. The compounds were individually identified on the basis of their retention time and molecular weight. The following 7 major flavonols were identified: compound 1, quercetin 3,7,4′-*O*-triglucoside (*m/z* = 787.1421); compound 2, quercetin 7,4′-*O*-diglucoside (*m/z* = 625.1396); compound 3, quercetin 3,4′-*O*-diglucoside (*m/z* = 625.1398); compound 4, isorhamnetin 3,4′-*O*-diglucoside (*m/z* = 639.1559); compound 5, quercetin 3-*O*-glucoside (*m/z* = 463.0886), compound 6, quercetin 4′-*O*-glucoside (*m/z* = 463.0873); compound 7, isorhamnetin 4-*O*’-glucoside (*m/z* = 477.1040). With respect to the PDA, the following maximum absorbance wavelengths were observed for each flavonol: quercetin 3,7,4′-*O*-triglucoside (346.7 nm), quercetin 7,4′-*O*-diglucoside (371.8 nm), quercetin 3,4′-*O*-diglucoside (343.2 nm), isorhamnetin 3,4′-*O*-diglucoside (346.7 nm), quercetin 3-*O*-glucoside (346.7 nm), quercetin 4′-*O*-glucoside (362.3 nm), and isorhamnetin 4′-*O*-glucoside (371.1 nm). The identified compounds coincide unequivocally with those reported by other authors in the bibliography [10,33,34,35]. The data corresponding to maximum absorbance wavelengths, mass spectra, theoretical and measured masses, and time of elution were included as Supplementary Material (Table S2). Once this preliminary identification has been carried out, the chromatographic method subsequently developed can be applied to other onion varieties, taking into account the retention times and absorbance spectra, without the need to apply masses. This is of interest because of the more time and effort involved in mass spectrometry and because it is not usually available for most food quality industries and research groups.

### 3.5. Separation and Quantification of Flavonols by UHPLC-PDA

The major flavonols found in the onions were separated and quantified by UHPLC by means of an ACQUITY UPLC H-Class system coupled to an ACQUITY UPLC Photodiode Array (PDA) detector. The PDA detector was set in the wavelength range of 210–400 nm for the 3D scan, with a data collection rate of 40 pts s^−1^. However, all the flavonols were quantified by 2D scan, and the PDA was set at 360 nm, which is approximately the maximum absorbance wavelength of quercetin 3-*O*-glucoside, i.e., the commercially available flavonol standard. Since the rest of the standards were not available, the rest of the compounds were quantified according to the calibration curve of quercetin 3-*O*-glucoside, on the basis of the structural similarities between these molecules while taking into account their molecular weights.

The flavonols were separated by injecting a 3.0 µL sample into an Acquity UPLC BEH C18 column (50 mm × 2.1 mm i.d., 1.7 mm particle size, Waters Corporation, Milford, MA, USA). The temperature was set at 55 °C and the mobile phase was a binary solvent system. Phase A was formed by 2% acetic acid in water and phase B by 2% acetic acid in acetonitrile. These conditions were selected in accordance with the expertise acquired by our research group from previous studies on this type of compounds [36,37,38]. The system was controlled by Empower3 Chromatography Data Software (Waters Corporation, Milford, MA, USA). The integration of flavonols was performed manually in the form ‘‘Valley-to-Valley”.

### 3.6. Box–Behnken Design (BBD)

A BBD design was employed to determine the effect of 3 factors (the 3 independent variables) on the UHPLC-PDA separation. The factors studied were flow rate (X_1_), solvent composition (% phase B) at the beginning of the gradient program (X_2_), and solvent composition (% phase B) at the end of the gradient program (X_3_). As these factors have different units and ranges, each factor was first normalized and forced into the range −1 to +1 [20]. Thus, a Box–Behnken design with 3 factors and 3 levels for each factor: low (−1), medium (0), and a high level (1), were set. Specifically, the studied ranges were as follows: flow rate (mL min^−1^): 0.4, 0.5, 0.6; %B at the beginning (%): 0, 5, 10; and %B at the end (%): 50, 75, 100. The ranges for the study were selected on the basis of our team previous experience. The analysis time between the beginning and the end of the gradient was 5 min. The response variables (dependent variables) were optimized according to the peak resolutions (Y_Rs_) and the analyses run time (Y_RT_). The retention time of the last peak in the chromatogram was regarded as the analysis run time, while the peak resolutions were calculated according to Equation (6).

(6) $$Rs=\frac{2\;\left(t_{R\left(B\right)}-t_{R\left(A\right)}\right)}{W_{b\left(A\right)}+W_{b\left(B\right)}}\;,$$

where *t_R (A)_* and *t_R (B)_* are the retention times of two adjacent peaks *A* and *B*, respectively, and *W_b (A)_* and *W_b (B)_* are the peak widths at the base of the same adjacent peaks. All of these parameters were calculated by means of Empower 3 Software (Waters Corporation, Milford, MA, USA).

On the basis of the number of factors and using the specific BBD equation, we obtained a design consisting of 15 experiments including 3 repetitions at their center point. The results obtained from the whole experimental design matrix were analyzed by response surface methodology. A mathematical model (Equation (7)) for each response can be built in which each response of the system was considered as a function of the corresponding factors and their corresponding interactions.

(7) $$y=\beta_{0}+\sum_{i=1}^{k}\beta_{i}X_{i}+\sum_{i=1}^{k}\;\beta_{ii}\cdot X_{i}^{2}+\sum_{i}\sum_{i=1}^{k}\beta_{ij}X_{ij}+\varepsilon ,$$

where *y* is the dependent variable; *X_i_* and *X_j_* are independent variables; *β_0_* is the regression coefficient for the intercept; *β_i_*, *β_ii_*, and *β_ij_* are the regression coefficients for the linear, quadratic, and interactive terms, respectively; and *ε* is the error.

In order to find the optimum chromatographic conditions for the 3 responses (peak resolutions and analysis run time) at the same time, we performed an MRO together with their desirability functions. Firstly, the data were analyzed to generate a separate model for each response. Then, the predicted values obtained from each response surface were transformed into an individual desirability function, *d_i_*. The scale of the desirability function ranges from 0 (for an unacceptable response value) to 1 (for a completely desirable one) [39]. *D* was calculated by combining the individual desirability values by applying the geometric mean (Equation (8)):

(8) D=(d1 × d2 ×…dm)1m,

where *d_i_* indicates the desirability of the responses and *m* is the number of responses in the measure. The MRO methodology is a very useful tool for quality control and analytical laboratories since it can minimize analyses costs and time.

### 3.7. Validation Procedure and Chromatographic Properties

Once the UHPLC method had been developed for the determination of the major flavonols in onions, it was validated according to the recommendations of ICH Guideline Q2 (R1) [27] and then their chromatographic characteristics were measured.

For the validation of the method, we evaluated linearity, precision (repeatability and intermediate precision), robustness, and detection (LODs) and quantification limits (LOQs). The calculations were performed by means of Microsoft Office Excel 2013. A calibration curve for quercetin 3-*O*-glucoside, the commercially available standard, was plotted. The linearity of the calibration curve was evaluated by calculating its coefficient of determination (*R*^2^). Since the rest of the standards were not available, these compounds were quantified according to the calibration curve of quercetin 3-*O*-glucoside, on the basis of the structural similarities between these molecules, while taking into account their molecular weights. The LODs and LOQs were obtained by dividing, respectively, 3 and 10 times the signal-to-noise ratios by the slope of the calibration curves that had been obtained. The repeatability and intermediate precision of the developed UHPLC method were evaluated according to the retention time and peak resolution of each compound. For this purpose, 9 replicates were completed on the same day. Regarding the intermediate precision, 21 replicates were completed on 3 different days. The precision was expressed as the coefficient of variance (CV) of each one of the above-mentioned parameters, where the acceptable CV limit was under 10%, in accordance with the AOAC manual for the Peer-Verified Methods program [28]. Finally, the robustness of the UHPLC method was also evaluated. For this purpose, specific variations in the range of the column temperature, the flow rate, and the injection volume were tested. For each parameter, 3 different variation levels were evaluated, and for each level, 4 repetitions were carried out. For the statistical analysis, two-tailed *t*-test assuming equal variances and a level of significance of 0.05 was employed.

With regards to the chromatographic properties, the retention time (*t_R_*), the resolution (Rs), the retention factor (*k*) (Equation (9)), and the selectivity (α) (Equation (10)) were evaluated.

(9) $$k=\frac{{t^{\prime }}_{R}}{t_{M}},$$

(10) $$\propto =\frac{{t^{\prime }}_{R}\left(B\right)}{{t^{\prime }}_{R}\left(A\right)}=\frac{K\left(B\right)}{K\left(A\right)},$$

where *t_R_*_(*A*)_ and *t_R_*_(*B*)_ are the retention times of two adjacent peaks *A* and *B*, respectively; *t_M_* is the column dead time; and t’_R_ is the adjusted retention time (*t’_R_* = *t_R_* − *t_M_*). All of these parameters were calculated by means of Empower 3 Software (Waters Corporation, Milford, MA, USA).

## 4. Conclusions

A rapid and reproducible UHPLC-PDA methodology for the separation of the seven major flavonols that can be found in onions was developed. A BBD was used in conjunction with MRO to optimize the simultaneous separation of the seven flavonols of interest. The optimal conditions for a successful separation were 9.9% phase B at the beginning of the gradient, 53.2% phase B at the end of the gradient, 55 °C column working temperature, and 0.6 mL min^−1^ flow rate. The analysis time between the beginning of the gradient and the end of the gradient was 5 min. The complete separation was achieved in less than 2.7 min with excellent chromatographic characteristics (resolution, selectivity, and retention factor). The method has also been successfully validated, showing high repeatability and intermediate precision values (CV < 5%) for retention time and peak resolution, low limits of detection and quantification, as well as good linearity. Furthermore, the robustness of the methodology was satisfactorily tested against variations of injection volume, temperature column, and flow rate for most of the flavonols studied. The developed methodology saves time, solvent, and costs in comparison to other methods found in the bibliography. The method was also applied to the analysis of yellow, white, and red onions to successfully demonstrate its applicability to the analysis of different onion varieties. This method would allow laboratories, researchers, and companies to determine onion flavonol contents in a particularly short time, which in turn would contribute to improving the quality of onions and their sub-products when targeted to human consumption.

## Figures and Tables

**Table 1:** Box–Behnken design matrix and experimental values obtained for resolutions and run time.

Run	Factors			Responses				
	X_1_	X_2_	X_3_	Rs_3–4_	Rs_4–5_	Rs_5–6_	Rs_6–7_	Run Time (min)
1	0	0	0	1.50	3.62	3.34	1.74	2.761
2	1	0	1	1.22	3.68	2.72	1.31	2.326
3	0	0	0	1.77	4.53	4.10	2.02	2.775
4	0	−1	1	1.08	2.46	2.12	1.22	2.658
5	−1	0	−1	2.01	3.93	3.68	2.20	3.574
6	−1	0	1	1.00	2.83	2.79	1.27	2.659
7	0	1	1	1.18	3.53	3.08	1.47	2.210
8	0	0	0	1.36	3.62	2.89	1.41	2.759
9	0	1	−1	2.35	5.40	4.81	2.83	2.924
10	1	−1	0	1.25	3.60	3.49	1.75	2.892
11	0	−1	−1	1.80	3.88	4.01	2.35	3.699
12	−1	−1	0	1.13	3.38	3.05	1.61	3.242
13	−1	1	0	1.49	3.76	3.01	1.58	2.705
14	1	1	0	1.82	4.30	3.63	1.88	2.298
15	1	0	−1	2.02	4.33	4.91	2.92	3.199

**Table 2:** Chromatographic properties of the developed ultra-high performance liquid chromatography (UHPLC) method for flavonols in onions.

Peak	Compounds	t_R_ (min)	Width (W_b_)	t’_R_	Retention Factor (*k*)	Selectivity (α)	Resolution (R_s_)
1	Quercetin 7,3,4′-*O*-triglucoside	0.945	2.85	0.448	1.36	-	-
2	Quercetin 7,4′-*O*-diglucoside	1.478	3.45	1.084	2.69	1.98	7.69
3	Quercetin 3,4′-*O*-diglucoside	1.688	4.10	1.174	3.22	1.19	2.15
4	Isorhamnetin 3,4′-*O*-diglucoside	1.835	2.40	1.315	3.59	1.11	1.93
5	Quercetin 3′-*O*-glucoside	2.119	2.55	1.599	4.30	1.20	4.56
6	Quercetin 4′-*O*-glucoside	2.478	5.65	1.951	5.19	1.21	3.68
7	Isorhamnetin 4′-*O*-glucoside	2.693	3.35	2.157	5.73	1.10	2.23

**Table 3:** Validation of the developed UHPLC method.

Peak	Compounds	Linear Equation	*R* ^2^	LOD (mg L^−1^)	LOQ (mg L^−1^)	CV ^1^ for Repeatability (%)		CV ^1^ for Intermediate Precision (%)	
						t_R_	Rs	t_R_	Rs
1	Quercetin 7,3,4′-*O*-triglucoside	y = 5069.90x + 8282.83	0.9997	0.0437	0.1454	2.33	-	2.80	-
2	Quercetin 7,4′-*O*-diglucoside	y = 6382.50x + 8282.83	0.9997	0.0347	0.1155	1.20	1.88	1.93	4.13
3	Quercetin 3,4′-*O*-diglucoside	y = 6382.52x + 8282.83	0.9997	0.0347	0.1155	1.17	3.89	1.91	4.59
4	Isorhamnetin 3,4′-*O*-diglucoside	y = 6247.89x + 8282.83	0.9997	0.0354	0.1181	0.95	2.52	1.76	2.72
5	Quercetin 3′-*O*-glucoside	y = 8610.35x + 8282.83	0.9997	0.0257	0.0857	0.96	4.88	1.70	4.90
6	Quercetin 4′-*O*-glucoside	y = 8610.35x + 8282.83	0.9997	0.0257	0.0857	0.88	1.53	3.17	3.65
7	Isorhamnetin 4′-*O*-glucoside	y = 8358.38x + 8282.83	0.9997	0.0265	0.0883	0.78	4.14	1.39	4.03

^1^ CV: coefficient of variation.

**Table 4:** Robustness of the UHPLC method developed.

		Column Temperatures (°C)			Flow Rates (mL min^−1^)			Injection Volumes (µL)		
		52	55	58	0.57	0.60	0.63	2.8	3.0	3.2
Retention time (min)	Quercetin 7,3,4′-*O*-triglucoside	1.026 ^a^	0.945 ^b^	0.882 ^c^	1.026 ^a^	0.945 ^b^	0.912 ^c^	0.955 ^a^	0.945 ^a^	0.929 ^a^
	Quercetin 7,4′-*O*-diglucoside	1.501 ^a^	1.477 ^a^	1.497 ^a^	1.590 ^a^	1.477 ^b^	1.540 ^b^	1.530 ^a^	1.477 ^a^	1.559 ^a^
	Quercetin 3,4′-*O*-diglucoside	1.797 ^a^	1.687 ^b^	1.679 ^b^	1.748 ^a^	1.687 ^b^	1.627 ^c^	1.694 ^a^	1.687 ^a^	1.674 ^a^
	Isorhamnetin 3,4′-*O*-diglucoside	1.936 ^a^	1.835 ^b^	1.804 ^b^	1.887 ^a^	1.835 ^b^	1.775 ^c^	1.839 ^a^	1.835 ^a^	1.823 ^a^
	Quercetin 3′-*O*-glucoside	2.231 ^a^	2.119 ^b^	2.002 ^c^	2.161 ^a^	2.119 ^b^	2.056 ^c^	2.126 ^a^	2.119 ^a^	2.111 ^a^
	Quercetin 4′-*O*-glucoside	2.591 ^a^	2.478 ^b^	2.357 ^c^	2.512 ^a^	2.478 ^a^	2.417 ^b^	2.484 ^a^	2.478 ^a,b^	2.471 ^b^
	Isorhamnetin 4′-*O*-glucoside	2.797 ^a^	2.693 ^a^	2.574 ^b^	2.721 ^a^	2.692 ^b^	2.633 ^c^	2.697 ^a^	2.693 ^a,b^	2.683 ^b^
Peak area	Quercetin 7,3,4′-*O*-triglucoside	1584 ^a^	1749 ^a^	1992 ^a^	1815 ^a^	1749 ^a^	1846 ^a^	1493 ^a^	1749 ^a^	2270 ^b^
	Quercetin 7,4′-*O*-diglucoside	4073 ^a^	4459 ^a^	5070 ^a^	5733 ^a^	4459 ^a^	5004 ^a^	3246 ^b^	4459 ^a,b^	5251 ^b^
	Quercetin 3,4′-*O*-diglucoside	104,048 ^a^	112,705 ^a^	123,675 ^a^	123,884 ^a^	112,705 ^a^	122,463 ^a^	88,187 ^a^	112,705 ^a^	140,119 ^b^
	Isorhamnetin 3,4′-*O*-diglucoside	5073 ^a^	5245 ^a^	5931 ^a^	5892 ^a^	5245 ^a^	5800 ^a^	4233 ^a^	5245 ^a^	6905 ^b^
	Quercetin 3′-*O*-glucoside	5045 ^a^	4899 ^a^	5396 ^a^	5330 ^a^	4899 ^a^	5448 ^a^	4015 ^a^	4899 ^a,b^	5811 ^b^
	Quercetin 4′-*O*-glucoside	151,175 ^a^	162,031 ^a^	178,379 ^a^	175,285 ^a^	159,754 ^a^	173,719 ^a^	130,086 ^a^	165,384 ^a^	198,383 ^b^
	Isorhamnetin 4′-*O*-glucoside	18,933 ^a^	20,539 ^a^	22,383 ^a^	22,187 ^a^	20,539 ^a^	21,882 ^a^	16,308 ^a^	20,539 ^a^	25,220 ^b^
Peak resolution	Quercetin 7,3,4′-*O*-triglucoside	-	-	-	-	-	-	-	-	-
	Quercetin 7,4′-*O*-diglucoside	6.22 ^a^	7.69 ^a^	6.94 ^a^	6.95 ^a^	7.69 ^a^	7.68 ^a^	7.62 ^a^	7.69 ^a^	8.15 ^a^
	Quercetin 3,4′-*O*-diglucoside	2.89 ^a^	2.15 ^a,b^	1.6 ^b^	1.62 ^a^	2.15 ^a^	0.810 ^b^	1.80 ^a^	2.15 ^b^	1.20 ^a^
	Isorhamnetin 3,4′-*O*-diglucoside	1.92 ^a^	1.93 ^a^	1.59 ^a^	2.09 ^a^	1.93 ^a^	1.95 ^a^	2.10 ^a^	1.93 ^a^	1.94 ^a^
	Quercetin 3′-*O*-glucoside	4.54 ^a^	4.56 ^a^	2.9 ^b^	4.60 ^a^	4.72 ^a^	4.56 ^a^	4.48 ^a^	4.56 ^a^	4.44 ^a^
	Quercetin 4′-*O*-glucoside	3.30 ^a^	3.68 ^a^	3.47 ^a^	3.70 ^a^	3.67 ^a^	3.68 ^a^	3.43 ^a^	3.68 ^a^	3.82 ^a^
	Isorhamnetin 4′-*O*-glucoside	1.87 ^a^	2.23 ^b^	2.11 ^a,b^	2.14 ^a^	2.23 ^a^	2.15 ^a^	2.10 ^a^	2.23 ^a^	2.16 ^a^

Same letter in the same row means that there were no significant differences as per the *t*-test (*p* < 0.05).

**Table 5:** Quantification of flavonols in white, yellow, and red onion varieties.

	Flavonol Composition (mg/10 g DW) ^1^							
	Quercetin 7,3,4′-*O*-triglucoside	Quercetin 7,4′-*O*-diglucoside	Quercetin 3,4′-*O*-diglucoside	Isorhamnetin 3,4′-*O*-diglucoside	Quercetin 3′-*O*-glucoside	Quercetin 4′-*O*-glucoside	Isorhamnetin 4′-*O*-glucoside	Total
Spring white onion 1	0.09 ± 0.00	0.62 ± 0.00	14.31 ± 0.19	0.56 ± 0.00	0.56 ± 0.00	11.74 ± 0.14	1.11 ± 0.02	28.98
Sweet white onion 2	0.08 ± 0.01	0.12 ± 0.00	5.61 ± 0.06	0.23 ± 0.00	0.43 ± 0.02	2.50 ± 0.07	0.20 ± 0.00	9.17
Spring white onion 3	0.15 ± 0.00	0.51 ± 0.00	9.17 ± 0.06	0.48 ± 0.01	0.47 ± 0.00	7.36 ± 0.04	0.73 ± 0.01	18.88
Sweet white onion 4	0.11 ± 0.00	0.11 ± 0.03	5.58 ± 0.22	0.25 ± 0.00	3.77 ± 0.00	1.03 ± 0.10	0.40 ± 0.02	11.23
Sweet white onion 5	0.06 ± 0.03	0.12 ± 0.02	8.41 ± 0.15	0.12 ± 0.01	0.45 ± 0.00	9.42 ± 0.09	0.84 ± 0.00	19.42
Sweet white onion 6	0.07 ± 0.00	0.10 ± 0.01	7.28 ± 0.07	0.21 ± 0.00	0.12 ± 0.00	6.61 ± 0.05	0.98 ± 0.00	15.36
Yellow onion 1	0.21 ± 0.02	0.53 ± 0.03	12.32 ± 0.02	0.46 ± 0.02	0.52 ± 0.00	15.47 ± 0.00	0.65 ± 0.01	30.17
Yellow onion 2	0.29 ± 0.01	0.10 ± 0.02	7.13 ± 0.13	0.52 ± 0.03	0.40 ± 0.12	5.18 ± 0.01	0.91 ± 0.02	14.53
Yellow onion 3	0.18 ± 0.00	0.22 ± 0.01	8.13 ± 0.02	0.32 ± 0.00	5.00 ± 0.01	1.37 ± 0.05	0.13 ± 0.00	15.35
Red onion 1	0.36 ± 0.02	0.88 ± 0.06	25.58 ± 0.16	0.72 ± 0.01	0.57 ± 0.02	18.42 ± 0.15	1.47 ± 0.00	48.00
Red onion 2	0.34 ± 0.03	0.69 ± 0.04	16.36 ± 0.04	0.83 ± 0.01	0.57 ± 0.00	18.69 ± 0.03	2.44 ± 0.00	39.92
Red onion 3	0.28 ± 0.00	0.56 ± 0.00	17.60 ± 0.05	0.52 ± 0.03	0.92 ± 0.01	21.94 ± 1.03	1.03 ± 0.01	42.86
Red onion 4	0.29 ± 0.02	0.86 ± 0.02	24.97 ± 0.32	0.83 ± 0.02	0.56 ± 0.00	16.73 ± 0.91	1.99 ± 0.06	46.23

^1^ Flavonoid composition expressed as mean of three replicates ± standard deviation (mg/10 g dry weight (DW) ± SD).

## Data Availability

The data presented in this study is contained within the article or supplementary material.

## Supplementary Materials

The following are available online at https://www.mdpi.com/article/10.3390/ph14040310/s1: Figure S1: Structural types of analyzed flavonols. Table S1: Characteristics of the different onions studied. Table S2: Mass spectra information for the seven flavonols identified in onion bulbs by UHPLC–photo-diode array (PDA)–quadrupole-time-of-flight mass spectrometry (Q-ToF-MS) in negative ESI mode.

## References

[1] Rodrigues A.S., Pérez-Gregorio M.R., García-Falcon M.S., Simal-Gándara J., Almeida D.P.F. (2010). Effect of post-harvest practices on flavonoid content of red and white onion cultivars. Food Control.

[2] FAO (2016). Global Forest Resources Assessment 2015.

[3] Block E. (1992). The Organosulfur Chemistry of the Genus *Allium*—Implications for the Organic Chemistry of Sulfur. Angew. Chem. Int. Ed. Engl..

[4] Özcan M.M., Süleyma D., Nurhan U. (2018). Effect of Species on Total Phenol, Antioxidant Activity and Phenolic Compounds of Different Wild Onion Bulbs. J. Food Meas. Charact..

[5] Ye C.L., Dai D.H., Hu W.L. (2013). Antimicrobial and antioxidant activities of the essential oil from onion (*Allium cepa* L.). Food Control.

[6] Park M.J., Ryu D.H., Cho J.Y., Ha I.J., Moon J.S., Kang Y.H. (2018). Comparison of the Antioxidant Properties and Flavonols in Various Parts of Korean Red Oni, ons by Multivariate Data Analysis. Hortic. Environ. Biotechnol..

[7] Ferioli F., D’Antuono L.F. (2016). Evaluation of Phenolics and Cysteine Sulfoxides in Local Onion and Shallot Germplasm from Italy and Ukraine. Genet. Resour. Crop Evol..

[8] Larson A.J., Symons J.D., Jalili T. (2010). Quercetin: A Treatment for Hypertension?—A Review of Efficacy and Mechanisms. Pharmaceuticals.

[9] Abian O., Ortega-Alarcon D., Jimenez-alesanco A., Ceballos-laita L., Vega S., Reyburn H.T., Velazquez-campoy A. (2020). Structural Stability of SARS-CoV-2 3CLpro and Identification of Quercetin as an Inhibitor by Experimental Screening. Int. J. Biol. Macromol..

[10] Pérez-Gregorio M.R., Regueiro J., González-Barreiro C., Rial-Otero R., Simal-Gándara J. (2011). Changes in Antioxidant Flavonoids during Freeze-Drying of Red Onions and Subsequent Storage. Food Control.

[11] Ko E.Y., Nile S.H., Jung Y.S., Keum Y.S. (2018). Antioxidant and Antiplatelet Potential of Different Methanol Fractions and Flavonols Extracted from Onion (*Allium cepa* L.). 3 Biotech.

[12] Jurinjak Tušek A., Benković M., Belščak Cvitanović A., Valinger D., Jurina T., Gajdoš Kljusurić J. (2016). Kinetics and Thermodynamics of the Solid-Liquid Extraction Process of Total Polyphenols, Antioxidants and Extraction Yield from Asteraceae Plants. Ind. Crop. Prod..

[13] Tistaert C., Dejaegher B., Heyden Y.V. (2011). Chromatographic Separation Techniques and Data Handling Methods for Herbal Fingerprints: A Review. Anal. Chim. Acta.

[14] Stipcovich T., Barbero G.F., Ferreiro-González M., Palma M., Barroso C.G. (2018). Fast Analysis of Capsaicinoids in Naga Jolokia Extracts (*Capsicum Chinense*) by High-Performance Liquid Chromatography Using Fused Core Columns. Food Chem..

[15] Moreno-Rojas J.M., Moreno-Ortega A., Ordóñez J.L., Moreno-Rojas R., Pérez-Aparicio J., Pereira-Caro G. (2018). Development and Validation of UHPLC-HRMS Methodology for the Determination of Flavonoids, Amino Acids and Organosulfur Compounds in Black Onion, a Novel Derived Product from Fresh Shallot Onions (*Allium Cepa* Var. Aggregatum). LWT-Food Sci. Technol..

[16] Park S.K., Jin D.E., Park C.H., Seung T.W., Guo T.J., Song J.W., Kim J.H., Kim D.O., Heo H.J. (2015). Ameliorating effects of ethyl acetate fraction from onion (*Allium cepa* L.) flesh and peel in mice following trimethyltin-induced learning and memory impairment. Food Res. Int..

[17] Lee S.U., Lee J.H., Choi S.H., Lee J.S., Ohnisi-Kameyama M., Kozukue N., Levin C.E., Friedman M. (2008). Flavonoid content in fresh, home-processed, and light-exposed onions and in dehydrated commercial onion products. J. Agric. Food Chem..

[18] Majid I., Vikas N. (2017). Instrumental Texture and FLavonoid Profile of Paste Developed from Sprouted Onion Varieties of Indian Origin. Int. J. Food Prop..

[19] González de Peredo A.V., Vázquez-Espinosa M., Piñeiro Z., Espada-Bellido E., Ferreiro-González M., Barbero G.F., Palma M. (2020). Development of a Rapid and Accurate UHPLC-PDA-FL Method for the Quantification of Phenolic Compounds in Grapes. Food Chem..

[20] Setyaningsih W., Saputro I.E., Carrera C.A., Palma M., Barroso C.G. (2017). Multiresponse optimization of a UPLC method for the simultaneous determination of tryptophan and 15 tryptophan-derived compounds using a Box-Behnken design with a desirability function. Food Chem..

[21] Wani T.A., Ahmad A., Zargar S., Khalil N.Y., Darwish I.A. (2012). Use of response surface methodology for development of new microwell-based spectrophotometric method for determination of atrovastatin calcium in tablets. Chem. Cent. J..

[22] Hu Z., Cai M., Liang H.H. (2008). Desirability Function Approach for the Optimization of Microwave-Assisted Extraction of Saikosaponins from Radix Bupleuri. Sep. Purif. Technol..

[23] Zheng J., Polyakova Y., Row K.H. (2006). Retention Factors and Resolutions of Amino Benzoic Acid Isomers with Some Lonic Liquids. Biotechnol. Bioproc. E.

[24] Sharma K., Assefa A.D., Kim S., Ko E.Y., Lee E.T., Park S.W. (2014). Evaluation of Total Phenolics, Flavonoids and Antioxidant Activity of 18 Korean Onion Cultivars: A Comparative Study. J. Sci. Food Agric..

[25] Turner C., Turner P., Jacobson G., Almgren K., Waldebäck M., Sjöberg P., Karlsson E.N., Markides K.E. (2006). Subcritical Water Extraction and β-Glucosidase-Catalyzed Hydrolysis of Quercetin Glycosides in Onion Waste. Green Chem..

[26] Snyder L.R., Dolan J.W. (2006). High-Performance Gradient Elution.

[27] ICH (2005). ICH Topic Q2 (R1) Validation of Analytical Procedures: Text and Methodology.

[28] AOAC (2012). AOAC Peer Verified Methods Program, Manual on Policies and Procedures.

[29] Pérez-Gregorio M.R., García-Falcón M.S., Simal-Gándara J., Rodrigues A.S., Almeida D.P.F. (2010). Identification and Quantification of Flavonoids in Traditional Cultivars of Red and White Onions at Harvest. J. Food Compos. Anal..

[30] Vallverdú-Queralt A., Medina-Remón A., Andres-Lacueva C., Lamuela-Raventos R.M. (2011). Changes in Phenolic Profile and Antioxidant Activity during Production of Diced Tomatoes. Food Chem..

[31] Vázquez-Espinosa M., Espada-Bellido E., González de Peredo A.V., Ferreiro-González M., Carrera C., Palma M., Barroso C.G., Barbero G.F. (2018). Optimization of Microwave-Assisted Extraction for the Recovery of Bioactive Compounds from the Chilean Superfruit (*Aristotelia Chilensis* (Mol.) Stuntz). Agronomy.

[32] González de Peredo A.V., Vázquez-Espinosa M., Espada-Bellido E., Ferreiro-González M., Amores-Arrocha A., Palma M., Barbero G.F., Jiménez-Cantizano A. (2019). Alternative Ultrasound-Assisted Method for the Extraction of the Bioactive Compounds Present in Myrtle (*Myrtus communis* L.). Molecules.

[33] Bonaccorsi P., Caristi C., Gargiulli C., Leuzzi U. (2008). Flavonol glucosides in *Allium* species: A comparative study by means of HPLC-DAD-ESI-MS-MS. Food Chem..

[34] Katsampa P., Valsamedou E., Grigorakis S., Makris D.P. (2015). A green ultrasound-assisted extraction process for the recovery of antioxidant polyphenols and pigments from onion solid wastes using Box–Behnken experimental design and kinetics. Ind. Crop. Prod..

[35] Tedesco I., Carbone V., Spagnuolo C., Minasi P., Russo G.L. (2015). Identification and Quantification of Flavonoids from Two Southern Italian Cultivars of *Allium cepa* L., Tropea (Red Onion) and Montoro (Copper Onion), and Their Capacity to Protect Human Erythrocytes from Oxidative Stress. J. Agric. Food Chem..

[36] de Souza Dias F., Lovillo M.P., Barroso C.G., David J.M. (2010). Optimization and validation of a method for the direct determination of catechin and epicatechin in red wines by HPLC/fluorescence. Microchem. J..

[37] Piñeiro Z., Palma M., Barroso C.G. (2004). Determination of Catechins by Means of Extraction with Pressurized Liquids. J. Chromatogr. A.

[38] Setyaningsih W., Saputro I.E., Carrera C.A., Palma M., García-Barroso C. (2019). Fast Determination of Phenolic Compounds in Rice Grains by Ultraperformance Liquid Chromatography Coupled to Photodiode Array Detection: Method Development and Validation. J. Agric. Food Chem..

[39] Maran J., Manikandan S., Thirugnanasambandham K., Vigna Nivetha C., Dinesh R. (2013). Box-Behnken Design Based Statistical Modeling for Ultrasound-Assisted Extraction of Corn Silk Polysaccharide. Carbohydr. Polym..

