# Supplementary material for: Development of a Rapid UHPLC-PDA Method for the Simultaneous Quantification of Flavonol Contents in Onions (Allium cepa L.)
Source: Pharmaceuticals (Basel). 2021 Apr 1;14(4):310. doi: 10.3390/ph14040310 (PMC8066725; doi:10.3390/ph14040310)
Supplement: Supplementary file 1 [file pharmaceuticals-14-00310-s001.pdf]

SUPPLEMENTARY MATERIAL

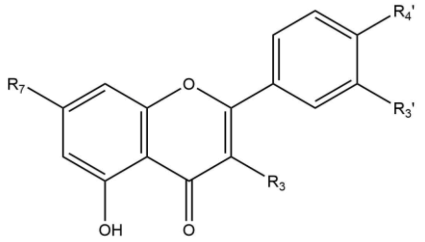

Flavonol type	R3	R7	R3′	R4′
Quercetin 3-*O*-glucoside	O-Glu	OH	OH	OH
Quercetin 4′-*O*-glucoside	OH	OH	OH	O-Glu
Quercetin 3,4′-*O*-diglucoside	O-Glu	OH	OH	O-Glu
Quercetin 7,4′-*O*-diglucoside	OH	O-Glu	OH	O-Glu
Quercetin 3,7,4′-*O*-triglucoside	O-Glu	O-Glu	OH	O-Glu
Isorhamnetin 4′-*O*-glucoside	OH	OH	O-CH3	O-Glu
Isorhamnetin 3,4′-*O*-diglucoside	O-Glu	OH	O-CH3	O-Glu

-O-Glu 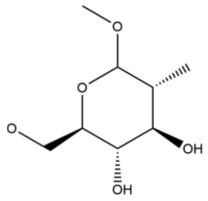

Figure S1. Structural types of analyzed flavonols.

Table S1. characteristics of the different onions studied.

Onion variety	Origin	Supermarket	Format	Company	Caliber (mm)
Spring white onion 1	Spain	DÍA	Sold by mesh net bag 2 kg	La Gramola	50/90
Sweet white onion 2	Chile	LIDL	Sold individually	-	-
Spring white onion 3	Spain	Mercadona	Sold 3 by 3	Tara	65
Sweet white onion 4	Spain	Mercadona	Sold by mesh net bag 2 kg	Tara	55
Sweet white onion 5	Spain	Carrefour	Sold in bulk	-	70/100
Sweet white onion 6	Spain	Carrefour	Sold by mesh net bag 500 g	Ajos Malsamar S.L.	60/80
Yellow onion 1	Spain (Valencia)	LIDL	Unit	-	-
Yellow onion 2	Spain	Carrefour	Sold in bulk	-	55/75 mm
Yellow onion 3	Spain	Carrefour	Sold 3 by 3. Assortment of yellow, red and white	-	60/80
Red onion 1	Spain	LIDL	Sold by mesh net bag 500 g	Linda	50/70 mm
Red onion 2	Austria	Mercadona	Sold by mesh net bag 500 g	Tara	50/90 mm
Red onion 3	Spain (Cuenca)	DÍA	Sold by mesh net bag 500 g	La Gramola	50/70 mm
Red onion 4	Spain (Castilla la Mancha/Valencia)	LIDL	Sold individually	-	-

Table S2. Mass spectra information for the seven flavonols identified in onion bulbs by UHPLC-PDA-Q-ToF-MS in negative ESI mode.

Peak number	Flavonols present in onion	Molecular formula	Maximum absorbance wavelength (nm)	Time of elution (min)	Theoretical mass [M-H]- (*m*/*z*)	Measured mass [M-H]- (*m*/*z*)
1	Quercetin 3,7,4′-*O*-triglucoside	$C_{33}H_{40}O_{22}$	346.7	2.873	787.1433	787.1421
2	Quercetin 7,4′-*O*-diglucoside	$C_{27}H_{30}O_{17}$	371.8	4.507	625.1425	625.1396
3	Quercetin 3,4′-*O*-diglucoside	$C_{27}H_{30}O_{17}$	343.2	5.138	625.1410	625.1398
4	Isorhamnetin 3,4′-*O*-diglucoside	$C_{28}H_{32}O_{17}$	346.7	5.213	639.1566	639.1559
5	Quercetin 3-*O*-glucoside	$C_{21}H_{20}O_{12}$	346.7	5.341	463.0877	463.0886
6	Quercetin 4′-*O*-glucoside	$C_{21}H_{20}O_{12}$	362.3	5.487	463.0882	463.0873
7	Isorhamnetin 4′-*O*-glucoside	$C_{22}H_{22}O_{12}$	371.1	5.543	477.1038	477.1040
